# Menopausal Vasomotor Symptoms and Subclinical Atherosclerotic Cardiovascular Disease: A Population‐Based Study

**DOI:** 10.1161/JAHA.123.033648

**Published:** 2024-08-21

**Authors:** Sigrid Nilsson, Angelika Qvick, Moa Henriksson, Sofia Sederholm Lawesson, Anna‐Clara Spetz Holm, Karin Leander

**Affiliations:** ^1^ Department of Obstetrics and Gynecology, and Department of Biomedical and Clinical Sciences Linköping University Linköping Sweden; ^2^ Unit of Cardiovascular and Nutritional Epidemiology Institute of Environmental Medicine, Karolinska Institutet Stockholm Sweden; ^3^ Department of Cardiology and Department of Health, Medicine and Caring Sciences Linköping University Linköping Sweden

**Keywords:** coronary atherosclerosis, epidemiology, vasomotor system, women's health, Epidemiology, Women, Risk Factors, Primary Prevention, Cardiovascular Disease

## Abstract

**Background:**

Menopausal vasomotor symptoms (VMS) are increasingly emphasized as a potentially important cardiovascular risk factor, but their role is still unclear. We assessed the association between VMS and subclinical atherosclerotic cardiovascular disease in peri‐ and postmenopausal women.

**Methods and Results:**

Using a cross‐sectional study design, questionnaire data were collected from a population‐based sample of women aged 50 to 64. The questionnaire asked whether menopause was/is associated with bothersome VMS. A 4‐point severity scale was used: (1) never, (2) mild, (3) moderate, and (4) severe. The VMS duration and time of onset were also assessed. Associations with subclinical atherosclerotic cardiovascular disease, detected via coronary computed tomography angiography, coronary artery calcium score, and carotid ultrasound were assessed using the outcome variables “any coronary atherosclerosis,” “segmental involvement score >3,” “coronary artery calcium score >100,” and “any carotid plaque,” using logistic regression. Covariate adjustments included socioeconomic, lifestyle, and clinical factors. Of 2995 women, 14.2% reported ever severe, 18.1% ever moderate, and 67.7% ever mild/never VMS. Using the latter as reference, ever severe VMS were significantly associated with coronary computed tomography angiography‐detected coronary atherosclerosis (multivariable adjusted odds ratio, 1.33 [95% CI, 1.02–1.72]). Corresponding results for ever severe VMS persisting >5 years or beginning before the final menstrual period were 1.50 (95% CI, 1.07–2.11) and 1.66 (95% CI, 1.10–2.50), respectively. No significant association was observed with segmental involvement score >3, coronary artery calcium score >100, or with any carotid plaque.

**Conclusions:**

Ever occurring severe, but not moderate, VMS were significantly associated with subclinical coronary computed tomography angiography‐detected atherosclerosis, independent of a broad range of cardiovascular risk factors and especially in case of long durations or early onset.

**Nonstandard Abbreviations and Acronyms**
CACS: coronary artery calcium score
MHT: menopausal hormone therapy
SCAPIS: Swedish CArdioPulmonary BioImage Study
SIS: segment involvement score
SWH: Study of Women's Health
VMS: vasomotor symptoms

**Clinical Perspective**

**What Is New?**

- To our knowledge, no previous study has used coronary computed tomography angiography in the analysis of the relationship between a history of vasomotor symptoms and the presence of subclinical atherosclerotic disease.
- A history of severe vasomotor symptoms was independent of a wide range of cardiovascular risk factors, significantly associated with coronary computed tomography angiography‐detected atherosclerosis, more pronounced when symptoms persisted >5 years or when they began before menopause.

**What Are the Clinical Implications?**

- Our results support previous research that has suggested a need for extra vigilance regarding cardiovascular risk factors in women suffering from severe vasomotor symptoms.

Atherosclerotic cardiovascular disease (ASCVD) is a major cause of mortality and morbidity in women.^1^ In addition to traditional cardiovascular risk factors, female reproductive‐related risk factors appear to play a significant role.^2^ To improve primary prevention strategies for ASCVD in women, there is a need for greater understanding of female‐specific risk factors that can be the focus of targeted preventive efforts.^3^, ^4^ Susceptibility to ASCVD in women increases considerably after menopause, when estrogen levels decline.^5^ The decline in estrogen levels is often accompanied by climacteric symptoms,^5^ such as hot flashes and night sweats, known as vasomotor symptoms (VMS), affecting approximately 80% to 97% of women^5^, ^6^ with varying frequency and severity.^6^, ^7^ The VMS duration is usually between 5 and 7 years,^8^, ^9^ but with an early onset the duration appears to be longer.^9^

Women with VMS appear to have an unfavorable cardiovascular risk profile; previous studies have found associations between VMS and elevated blood pressure, total cholesterol, and body mass index.^10^ Associations with sleep‐related problems and depressive symptoms have also been reported,^9^, ^11^ especially among women whose VMS persisted over time.^9^ Furthermore, associations with greater carotid intima media thickness have been demonstrated^12^, ^13^, ^14^, ^15^, ^16^ although overall results are divergent.^10^ Previous studies have also reported associations with carotid plaque,^13^ bloodflow velocity of the common carotid arteries,^12^ and brachial artery flow‐mediated dilatation.^15^, ^17^ Studies of VMS in relation to coronary artery calcium (CAC) have not shown significant associations^10^ whereas associations with aortic calcification have been demonstrated.^14^, ^17^

Among the previous studies of VMS in relation to subclinical CVD, a few considered VMS severity,^12^ frequency,^13^, ^16^ duration,^14^ or early time of onset^13^, ^18^ and found these to be significant for the association. Prospective studies of VMS in relation to risk of clinically diagnosed CVD^19^, ^20^ and CVD mortality^15^ have also reported significance of severity,^20^ frequency,^19^ persistence,^19^ and time of onset.^15^, ^20^

A number of different pathophysiological explanations for observed links between VMS and CVD have been proposed, including an overreactivity of the sympathetic nervous system, with inhibition of cardiac vagal control,^21^ and changes in the hypothalamic–pituitary–adrenal axis associated with variable cortisol concentrations.^22^, ^23^

To gain a better understanding of whether VMS are associated with ASCVD, population‐based studies with well‐characterized study participants are needed, allowing for detailed assessment of VMS as well as comprehensive adjustments for covariates. Using subclinical outcome measures has the advantage of investigating associations with early‐stage ASCVD when the opportunities for preventive interventions are greatest. Previous research addressing a possible association between VMS and subclinical ASCVD has been limited to the use of electron beam tomography and ultrasound measurements of the carotid and brachial arteries. The present study adds coronary computed tomography angiography (CCTA), a low dose imaging technique measuring coronary atherosclerosis with the ability to identify both the anatomical distribution and the characteristics of coronary atherosclerosis and plaques.^24^ CCTA data,^25^ CAC,^26^ and carotid plaque measurements^27^ have been shown to be good predictors of future CVD events in asymptomatic populations.

The aim of the present study was to assess associations between a self‐reported history of VMS, considering VMS severity, duration, and time of onset and subclinical ASCVD detected through CCTA, CAC, or carotid ultrasound.

## Methods

### Study Design and Population

Because of the sensitive nature of the data collected for this study, the data are not publicly available. Applications to access data may be sent to the SCAPIS (Swedish CArdioPulmonary BioImage Study) office at https://www.scapis.org/data‐access/.

This is a cross‐sectional substudy of the SCAPIS, a multicenter population‐based observational study aimed at improving prediction and prevention of CVD.^24^ The SCAPIS protocol, described in detail elsewhere,^24^ was consistent for all centers and included collection of data through a comprehensive questionnaire, anthropometry, ECG, blood pressure measurements, accelerometer measurements of physical activity, blood sampling, and extensive cardiovascular imaging.

Among the randomly selected Swedish men and women invited to participate in SCAPIS, 30 154 individuals were recruited (50.3%). The participation rate was similar for the sexes and across ages.^24^

Female study participants at 2 of the SCAPIS centers (Stockholm and Linköping) were invited to participate in the SWH (Survey on Women's Health) substudy, requiring completion of an additional questionnaire including questions about menopausal status, age at menstrual cessation, menopausal symptoms, and the use of menopausal hormone therapy (MHT) and naturally derived compounds. The questionnaire was first tested in a small pilot study and some adjustments for greater clarity were made.

Of the women invited to the SWH, 98% (n=5043) agreed to participate. Among those, 578 women gave no information about menopausal status and were therefore not considered. For most of this group, many other answers to reproduction‐related questions were also missing. The current study also has the following exclusion criteria: (1) being premenopausal (ie, regular menses during the past year), (2) loss of menstruation due to other causes than natural menopause (hormonal treatment, surgical menopause, excessive exercising or dietary restrictions, pregnancy, or breastfeeding), (3) missing data on VMS, (4) history of ischemic heart disease (definition given in Data S1), percutaneous coronary intervention or coronary artery bypass grafting (applies only to analyses of VMS in relation to coronary atherosclerosis), and (5) history of ischemic stroke (applies only to analyses of VMS in relation to carotid plaques). The exclusion criteria and sample selection methods for the SWH are visualized in Figure S1.

The SCAPIS and the SWH were approved by the Swedish Ethical Review Authority, reference numbers 2010‐228‐31M, 2015‐246/32, and 2020‐03101. Participants gave informed consent before taking part. The data collection took place between October 2015 and March 2018 in Stockholm and between October 2015 and June 2018 in Linköping. Objectively collected data on medical history and dispensed drugs were obtained from the National Patient Register and the National Prescribed Drug Register, through the individuals' unique personal identification number.

Parts of this work have been published to help fulfill the requirements for a doctoral degree at Linköping University in 2023.^28^

### Menopause and Vasomotor Symptoms

Menopausal status was defined according to the Stages of Reproductive Aging Workshop criteria^29^ as perimenopausal (3 months to <1 year amenorrhea), early postmenopausal (≥1–6 years amenorrhea), or late postmenopausal (>6 years amenorrhea). We categorized menopausal status based on the woman's response to questions about whether menses stopped (no menses in the past year) or partially stopped (menses stopped for at least 3 consecutive months in the past year). Women who reported ongoing use of MHT (n=281), which may mask bleeding patterns, were also categorized using this approach, although in this group there is some uncertainty about menopausal status. The SWH asked whether the women were currently or previously bothered by menopausal symptoms in the form of hot flushes or night sweats (here termed VMS), and if so to what degree and over what period(s) of time. A 4‐point severity scale was used, in accordance with the structure of the Greene Climacteric Scale,^30^ an established questionnaire to identify VMS: (1) never, (2) mild, (3) moderate, or (4) severe VMS. If a woman reported different degrees of VMS during different time periods, only the most severe degree was considered. In the cases of use of MHT, the instruction was to report VMS as they were before medication initiation. Information on age at onset of VMS and at possible cessation of VMS was also requested. Based on this information, the duration of VMS was determined. By comparing the age at onset of VMS with the age at final menstrual period (FMP), we determined whether the debut of VMS occurred pre‐FMP, at the same age as FMP, or post‐FMP. The SWH also included a question on frequency of VMS in the past 2 weeks, with 5 predefined response options: (1) no symptoms, (2) occasionally, (3) a few times per week, (4) once a day, and (5) several times per day. This information was used only for descriptive purposes, which also applied to information about the ever occurrence of other menopausal symptoms and the ever use of naturally derived herbal medicine as treatment (eg, phytoestrogens) to alleviate VMS.

### Coronary and Carotid Atherosclerosis Measures

CCTA data were collected using a multislice CT scanner (Siemens, Somatom Definition lash, Siemens Healthineers, Erlangen, Germany). Identical software and hardware was used at both study sites. The preparation phase for the CCTA imaging included detection of contraindications for the contrast media administration such as renal dysfunction or contrast media allergy. Before the procedure took place, a β‐blocker (metoprolol) and sublingual glyceryl nitrate were given to obtain adequate heart rate control and coronary artery relaxation to perform the imaging with good resolution. Iohexol (350 mg I/mL; GE Healthcare) at a dose of 325 mg I/kg body weight was used as the contrast medium with 2 different radiation intensities: 100 or 120 kV. Depending on heart rate, heart rate variability, presence of calcifications, and body weight, 1 of 5 separate imaging protocols was chosen. Additional details of image collection, processing, analyses, reconstruction, reading, and scoring are published elsewhere.^31^ Coronary atherosclerosis assessment data were reported in 18 segments,^32^ with findings from the most clinically relevant segments (1–3; 5–7; 9; 11–13; 17) mandatory to report. The coronary segments were visually examined for the presence of calcified or noncalcified plaques. The status of each segment was classified as “no atherosclerosis,” “nonsignificant (ie, 1%–49%) stenosis,” “significant (ie, ≥50%) stenosis,” “not assessable due to calcium blooming,” “not assessable due to technical failure,” or “segment missing.” A binary variable was created for presence or not of any CCTA‐detected atherosclerosis. Coronary segmental involvement score (SIS) was calculated by assigning 1 point to each segment where atherosclerosis was identified. A binary variable was created using a cutoff SIS score value of >3.

The CAC images were acquired using ECG‐gated noncontrast CT imaging at 120kV. Two different CAC protocols were available; a flash spiral protocol was used for subjects with a body weight <90 kg and a regular heart rate, whereas for all others a sequential protocol was used. All CAC images were reconstructed (B35f HeartView medium CaScore) and CAC was scored using the syngo calcium scoring software (Volume Wizard, Siemens, Forchheim, Germany). Lesions exceeding the calcium threshold of 130 Hounsfield units in at least 3 neighboring pixels per 1 mm^3^ were identified with 3‐dimensional‐based picking and viewing tools. A CAC score (CACS) was calculated after summing the calcium content in each coronary artery according to Agatston.^33^ CACS was dichotomized with 2 different cutoff values: >100 (versus 0–100) and >0 (versus 0) Agatston units.

The carotid arteries were assessed bilaterally for atherosclerotic plaques in the common carotid artery, bulb, and the internal carotid artery. Results were categorized as no, unilateral, or bilateral plaques. A binary variable for any plaque was created where the presence of either unilateral or bilateral plaques was contrasted with no plaques. Siemens Acuson S2000 ultrasound scanner equipped with a 9L4 linear transducer (Siemens Healthineers) was used according to a standardized protocol. Two‐dimensional grayscale images were produced and examined to evaluate the size and number of plaques in left and right carotid artery and intima‐media thickness.^34^, ^35^ Plaques were defined as focal structures encroaching into the arterial lumen by at least 0.5 mm, or 50% of the surrounding intima‐media thickness value or demonstrating a thickness >1.5 mm as measured from the intima‐lumen interface to the media‐adventitia interface.

The primary measures of subclinical ASCVD in the present study were (1) any coronary atherosclerosis, (2) SIS >3, (3) CACS >100, and (4) any carotid plaque.

### Covariates

The details of the physical examinations, anthropometry, and the collection and analyses of blood samples have previously been described.^31^ Based on questionnaire data, binary variables were created for educational level (university level versus lower), country of birth (Sweden versus outside Sweden), menopausal status (peri‐ versus postmenopausal), employment status (working versus nonworking), financial strain (difficulties versus nondifficulties managing regular expenses the past 12 months), sleep quality (usually bad or very bad sleep versus usually normal, good, or very good sleep), continuous stress (experience of continuous stress last year or past 5 years versus no experience of stress or only for some periods in life including the past 5 years), depression (during the past 12 months feeling sad, blue, or depressed for 2 weeks or more in a row, and responding “yes” to 5 or more out of 7 questions designed to capture depressive symptoms, versus not fulfilling these criteria), high alcohol consumption (consumption according to the upper half of an established 4‐point scale generated from responses to a set of 10 separate questions), and parental history of myocardial infarction and stroke (father or mother suffering a myocardial infarction or stroke before the ages of 60 and 65, respectively). The participants were categorized as current, previous, or never smokers. Among ever smokers, the sum of cigarette pack‐years was calculated. Data on specific medical diagnoses (ischemic heart disease, hypertension, hyperlipidemia, ischemic stroke, diabetes, sleep apnea, preeclampsia, gestational hypertension, and gestational diabetes) were derived from the questionnaire and from national registers. Accelerometry was used to measure physical activity and sedentary time. Study participants wore a triaxial accelerometer (ActiGraph LCC, Pensacola, FL) on their right hip during waking hours for 7 consecutive days. The ActiLife v.6.13.3 software was used to set up, download, and process the data. Data were registered as counts per minute (cpm), with higher cpm indicating more intense physical activity. Based on percentage of all wear time, physical activity intensities were categorized into (1) sedentary, 0 to 199 cpm, (2) light physical activity, 200 to 2689 cpm, and (3) moderate to vigorous, ≥2690 cpm, in accordance with previous studies.^36^ For descriptive purposes, the proportion of time spent in each physical activity intensity category of all wear time was also calculated. Further details about variable definitions are given in Data S1.

Past and current use of prescription systemic MHT, either in the form of oral pills, transdermal patches, or transdermal gels and irrespective of indication, were requested via questionnaire. A categorical variable was created considering duration of use: (1) never use, (2) 1 to 4 years, and (3) ≥5 years. In this classification, no differentiation was made between current and previous use of MHT.

### Statistical Analysis

The independent *t* test, the Mann–Whitney *U* test, or the chi‐square test, as appropriate, were used to assess statistical significance in comparisons across groups. Multivariate logistic regression models were applied to assess associations between a history of VMS and subclinical ASCVD, with results presented as odds ratios (ORs) with 95% CIs. Women who had never experienced VMS or only mild VMS (herein referred to as the “ever mild/never VMS group”) served as the reference and were contrasted with ever moderate and ever severe VMS, respectively.

We categorized the VMS variables under study as follows. A binary variable was created that distinguished longer from shorter duration of VMS. As there is no established cutoff to make such a distinction, we chose a cutoff corresponding to the median VMS duration in the study sample. Then a variable was created with the following categories: (1) ever mild/never VMS (reference), (2) ever moderate VMS ≤5 years, (3) ever moderate VMS >5 years, (4) ever severe VMS ≤5 years, and (5) ever severe VMS >5 years. Correspondingly, a variable differentiating pre‐FMP from post‐FMP VMS onset was created with the following categories: (1) ever mild/never VMS (reference category), (2) ever moderate VMS starting at age for FMP or at a younger age, (3) moderate VMS starting at a higher age than age at FMP, (4) severe VMS starting at age for FMP or at a younger age, and (5) severe VMS starting at a higher age than age at FMP.

In addition to a crude model, 4 multivariate models were constructed: model 1, age at time of study inclusion and site; model 2, the variables in model 1 plus educational level, country of birth, menopausal status, systolic blood pressure, waist circumference, low‐density lipoprotein cholesterol, triglycerides, diabetes, medically treated hyperlipidemia, medically treated hypertension, smoking status, and moderate to vigorous physical activity; model 3, the variables in model 2 plus continuous stress, quality of sleep, depression, and sleep apnea; and model 4, the variables in model 3 plus MHT.

In supplemental analyses of VMS severity in relation to coronary atherosclerosis, women with ever mild VMS served as the reference category, whereas women who had never experienced VMS constituted a separate category. Supplemental analyses were also performed to assess VMS severity in relation to CACS >0. Finally, a sensitivity analysis was performed excluding women with MHT initiation in late postmenopause (>6 years after the FMP), as well as one in which women diagnosed with ischemic heart disease were also included.

Statistical tests were 2 sided and *P* values <0.05 were considered to indicate statistical significance. All analyses were performed with SPSS v.28.0 (IBM, Portsmouth, UK).

## Results

### Descriptive Characteristics

The mean age of the 4465 participants in the SWH population was 57.4 years; 82.6% were postmenopausal, 8.2% perimenopausal, and 9.2% premenopausal. Population characteristics and prevalence of subclinical ASCVD by menopausal status are presented in Tables S1–S3. After excluding participants according to the specified criteria, 2995 women remained and constituted the study population (Figure S1). Of these, 43.3% reported ever occurrence of mild, 18.1% ever occurrence of moderate, and 14.2% ever occurrence of severe VMS whereas 24.4% had never experienced VMS. Postmenopausal women made up 89.7% of the study population, and the remaining women were perimenopausal. Of the postmenopausal women, 50.4% were late postmenopausal and 41.1% early postmenopausal, while 8.2% could not be categorized. Characteristics of the study population by severity of ever occurring VMS are presented in Tables 1 and 2. Women in the “ever severe VMS group” were more likely to be postmenopausal (93.4% versus 88.9%, *P*<0.01), and they had experienced other menopausal symptoms (42.1% versus 24.3%, *P*<0.01) and used herbal medicines (20.4% versus 5.5%, *P*<0.01) to a greater extent than women with ever mild/never VMS.

**Table 1:** General Characteristics by Vasomotor Symptom Category

Characteristics	Total study population n=2995	Ever mild/Never VMS n=2027 67.7%	Ever moderate VMS n=543 18.1%	Ever severe VMS n=425 14.2%
Age, y, mean (SD)	57.7 (4.2)	58.3 (4.2)	57.7 (4.3)†	58.4 (4.1)
Born outside Sweden, n (%)	357 (11.9)	230 (11.5)	53 (9.9)	74 (17.7)‡
University degree, n (%)	1473 (49.2)	1012 (50.7)	285 (53.3)	176 (42.1)†
Currently employed, n (%)	2488 (83.1)	1681 (83.9)	462 (86.4)	345 (82.3)
Financial strain, n (%)	108 (3.6)	62 (3.1)	18 (3.4)	28 (6.7)‡
Smoking status, n (%)				
Past	1167 (39.0)	753 (38.1)	217 (40.9)	197 (48.0)‡
Current	332 (11.1)	209 (10.6)	68 (12.8)	55 (13.4)
Pack years for cigarettes, median (25th–75th percentile)	11.0 (4.7–19.8)	10.5 (4.5–19.5)	11.8 (5.0–21.0)	13.3 (5.5–21.0)*
Medical history, n (%)				
Ischemic heart disease	48 (1.6)	39 (1.9)	5 (0.9)	4 (0.9)
Ischemic stroke	58 (1.9)	41 (2.0)	11 (2.0)	6 (1.4)
Hypertension	526 (17.6)	353 (17.4)	93 (17.1)	80 (18.8)
Hyperlipidemia	221 (7.4)	166 (8.2)	31 (5.7)	24 (5.6)
Diabetes	108 (3.6)	78 (3.8)	14 (2.6)	16 (3.8)
Sleep apnea	65 (2.2)	45 (2.2)	8 (1.5)	12 (2.8)
Parental history, n (%)				
Myocardial infarction	208 (6.9)	144 (7.2)	32 (6.0)	32 (7.7)
Stroke	196 (6.5)	126 (6.3)	30 (5.6)	40 (9.6)*
Overweight (25–29.9 kg/m^2^), n (%)	1116 (37.3)	711 (35.1)	224 (41.3)†	181 (42.6)†
Obese (≥30 kg/m^2^), n (%)	541 (18.1)	379 (18.7)	83 (15.3)	79 (18.6)
Waist circumference, cm, mean (SD)	89.1 (12.5)	89.0 (12.9)	88.8 (11.7)	89.6 (11.7)
Blood pressure, mm Hg				
Systolic blood pressure, mean (SD)	128 (18)	128 (18)	128 (18)	128 (19)
Systolic blood pressure >140, n (%)	719 (24.0)	490 (24.2)	127 (23.4)	102 (24.0)
Diastolic blood pressure, mean (SD)	80 (10)	81 (11)	81 (10)	80 (11)
Diastolic blood pressure >90, n (%)	575 (19.2)	399 (19.7)	98 (18.0)	78 (18.4)
Cardiometabolic biomarkers				
Low‐density lipoprotein cholesterol, mmol/L, mean (SD)	3.3 (0.9)	3.3 (1.0)	3.3 (0.9)	3.3 (1.0)
Triglycerides, mmol/L, mean (SD)	1.0 (0.6)	1.0 (5.3)	1.0 (0.5)	1.1 (0.7)†
High‐sensitivity C‐reactive protein, mg/L, median (25th–75th percentile)	0.9 (0.4–1.9)	0.9 (0.6–2.1)	0.9 (0.6–1.9)	1.1 (0.6–2.2)
Hemoglobin A1c, mmol/mol, mean (SD)	36.5 (5.6)	36.6 (5.9)	36.3 (3.9)	36.6 (5.7)
Bad or very bad sleep quality, n (%)	505 (16.9)	281 (14.2)	106 (20.1)†	118 (28.9)‡
Physical activity (% of all wear time), mean (SD)				
Sedentary	50.9 (9.8)	51.6 (10.0)	51.4 (9.7)	50.0 (9.7)
Moderate to vigorous activity	6.6 (3.4)	6.7 (3.4)	6.8 (3.3)	6.5 (3.6)
High alcohol consumption, n (%)	281 (9.4)	160 (8.9)	76 (15.4)‡	45 (12.3)
Continuous stress past 1–5 years, n (%)	751 (25.1)	463 (23.5)	155 (29.2)†	133 (32.6)‡
Depression, n (%)	671 (22.4)	405 (20.3)	146 (27.3)‡	120 (29.2)‡

Selection from the SWH in the SCAPIS. For all statistical comparisons, the “ever mild/never VMS group” is used as the reference category.

SCAPIS indicates Swedish Cardiopulmonary bioImage Study; SWH, Survey of Women's Health; and VMS, vasomotor symptoms.

* *P* < 0.05.

^†^ *P* < 0.01.

^‡^ *P* < 0.001.

**Table 2:** Reproduction‐Related Characteristics by Vasomotor Symptom Category

Characteristics	Total n=2995	Ever mild/never VMS n=2027 67.7%	Ever moderate VMS n=543 18.1%	Ever severe VMS n=425 14.2%
Age at menopause, y, mean (SD)	50.4 (3.7)	50.9 (3.6)	51.1 (3.5)	50.6 (4.1)
Time since menopause, y median (25th–75th percentile)	7 (3–10)	7 (4–11)	6 (3–9)	7 (4–11)
VMS§, n (%)				
Current	1173 (55.5)	642 (53.8)	296 (57.1)	235 (58.8)
Previous	938 (44.5)	551 (46.2)	222 (42.9)	165 (41.3)
Duration, y, median (25th–75th percentile)	4 (2–7)	4 (2–6)	5 (3–8)‡	6 (3–10)‡
Duration >5 years	820 (39.5)	331 (27.9)	289 (58.3)‡	200 (51.0)‡
Duration ≤5 years	1254 (60.5)	855 (72.1)	207 (41.7)‡	192 (49.0)‡
Age at onset, y, mean (SD)	49.8 (6.0)	50.5 (6.1)	49.9 (5.6)	49.4 (6.4)†
Onset before the age at FMPǁ	712 (39.6)	373 (37.4)	184 (42.0)	155 (42.9)
Onset at the age of FMP or olderǁ	1085 (60.4)	625 (62.6)	254 (58.0)	206 (57.1)
VMS frequency in women with current VMS§ n (%)				
Several times a day for the past 2 weeks	267 (22.8)	48 (7.5)	103 (34.8)‡	116 (49.4)‡
A few times a day for the past 2 weeks	267 (22.8)	139 (21.6)	82 (27.7)	46 (19.6)
A couple of times a week the past 2 weeks	190 (16.2)	119 (18.5)	50 (16.8)	21 (8.9)†
A few times in the past 2 weeks	325 (27.7)	250 (38.9)	46 (15.5)†	29 (12.3)‡
None in the past 2 weeks	125 (10.7)	87 (13.6)	15 (5.1)*	23 (9.8)
Ever bothered by other menopausal symptoms, n (%)	876 (29.2)	488 (24.3)	210 (38.9)‡	178 (42.1)‡
Menopausal hormone therapy				
Ever use n (%)	608 (22.9)	222 (10.9)	172 (31.7)‡	214 (50.3)‡
Age at initiation, y, mean (SD) Ever	51.0 (5.1)	51.6 (5.5)	51.4 (4.0)	50.9 (4.6)
Duration, years, median (25th–75th percentile)	4 (1–6)	3 (1–5)	3 (1–5)	5 (2–7)^‡^
Ever use of herbal medicine for VMS, n (%)	280 (9.3)	110 (5.5)	85 (16.0)‡	85 (20.4)‡
Medical obstetric history				
Preeclampsia	154 (5.1)	107 (5.3)	25 (4.6)	22 (5.2)
Gestational hypertension	58 (1.9)	43 (2.1)	11 (2.0)	4 (0.9)
Gestational diabetes	68 (2.3)	51 (2.5)	10 (1.9)	7 (1.7)

Selection from the SWH in the SCAPIS. For all statistical comparisons, the “ever mild/never VMS group” is used as the reference category.

FMP indicates final menstrual period; SCAPIS, Swedish Cardiopulmonary bioImage Study; SWH, Survey of Women's Health; and VMS, vasomotor symptoms.

* *P* < 0.05.

^†^ *P* < 0.01.

^‡^ *P* < 0.001.

^§^ Women who reported never experiencing VMS are not considered in the “ever mild/never VMS” column.

^ǁ^ Perimenopausal women are not considered.

VMS occurred earlier in women who reported ever severe VMS than in those who reported ever mild VMS (mean age 49.4 versus 50.5, *P*<0.01). In 39.6% of postmenopausal women who reported a history of VMS and provided information about time of VMS onset, symptoms began before the age at FMP. When compared with the group with ever mild VMS, such early onset of VMS was somewhat, although not significantly, more common in both the “ever moderate VMS” and “ever severe VMS” groups (Table 2). The duration of VMS was longer in both the “ever severe VMS” (median 6 years) and the “ever moderate VMS” group (median 5 years) than in the “ever mild VMS group” (median 4 years; *P*<0.01). In women with ongoing VMS at the time of the survey, the reported severity of VMS was clearly linked to reported frequency of VMS (Table 2).

Among women in the study population with current or previous MHT use, 91.6% started their treatment within 6 years of the FMP and the median duration of use was 4 years (interquartile range, 1–6 years). The majority (71%) reported use of oral treatment whereas 29% reported use of transdermal patches or gel. Women with ever severe or ever moderate VMS were more likely to have used MHT compared with the ever mild/never group (50.3 versus 10.9% and 31.7 versus 10.9%; *P*<0.01). In women with ever severe VMS, 24.9% had used MHT for ≥5 years, whereas the corresponding proportions in the “ever moderate VMS” and “ever mild VMS” group were 12.2% and 3.5% (Table 2).

Women in the “ever severe VMS” group were, compared with women in the “ever mild/never VMS” group, more often born outside Sweden, overweight, and past smokers. They also generally had a lower level of education and had more often suffered from financial strain in the past year. Further, they reported worse sleep quality, more depressive symptoms, and more continuous stress in the past 5 years. Neither physical activity, blood pressure, nor cardiometabolic biomarkers, except triglycerides, differed between the groups; triglycerides were significantly higher in the “ever severe VMS” group (Table 1).

The prevalence of any CCTA‐detected atherosclerosis was higher in the “ever severe VMS” group than in the “ever mild/never VMS” group (34.1 versus 27.8%, *P*=0.017). Similarly, CACS >0 was more prevalent in the “ever severe VMS” group than in the “ever mild/never VMS” group (34.4 versus 29.3%, *P*=0.014). For “ever moderate VMS” there were no corresponding differences. The prevalence of SIS >3, CACS >100, and carotid plaques did not differ significantly between groups (Table 3).

**Table 3:** Prevalence of Subclinical ASCVD Setected by CT, CCTA, or Ultrasound by Vasomotor Symptom Category

Measures	Total study population n=2995)	Ever mild/never VMS n=2027 67.7%	Ever moderate VMS n=543 18.1%	Ever severe VMS n = 425 14.2%
Coronary artery calcium score (AU), n (%)				
>0	858 (28.6)	578 (29.3)	137 (25.7)	143 (34.4)*
>100	184 (6.1)	135 (6.9)	25 (4.7)	24 (5.8)
CCTA, n (%)				
Any coronary atherosclerosis	781 (26.1)	516 (27.8)	130 (25.9)	135 (34.1)*
Segment involvement score >3	120 (4.0)	84 (4.5)	16 (3.2)	20 (5.1)
Any carotid plaque, n (%)	1399 (46.7)	942 (46.6)	255 (47.2)	202 (47.6)

Selection from the SWH in the SCAPIS. For all statistical comparisons, the ever mild/never VMS group is used as the reference category.

ASCVD indicates atherosclerotic cardiovascular disease; AU, Agatston units; CCTA, coronary computed tomography angiography; CT, computed tomography; SCAPIS, Swedish Cardiopulmonary bioImage Study; and SWH, Survey of Women's Health.

* *P* < 0.05.

Women who never experienced VMS were significantly older and had other cardiovascular risk factors to a greater degree than women with mild VMS (Tables S4–S6).

The proportion of missing data was <8% for the variables used in the regression models; in adjusted models, participants for whom relevant covariate data were incomplete were not considered. Variable‐specific information on missing data is provided in Table S7.

The characteristics of the excluded groups were generally similar to those of the included group (Tables S8 and S9).

### VMS in Relation to Subclinical ASCVD

Ever severe VMS, but not moderate, were significantly associated with coronary atherosclerosis in crude and adjusted models (Figure and Table S10). The OR point estimate generated in model 3, OR 1.33 (95% CI, 1.02–1.72) became somewhat higher when adjusting additionally for MHT (model 4), OR 1.42 (95% CI, 1.07–1.88). Excluding women with start of MHT in late postmenopause (n = 51) did not substantially affect this result (Table S11). Ever moderate VMS was not significantly associated with coronary atherosclerosis in any of the models.

**Figure 1:**
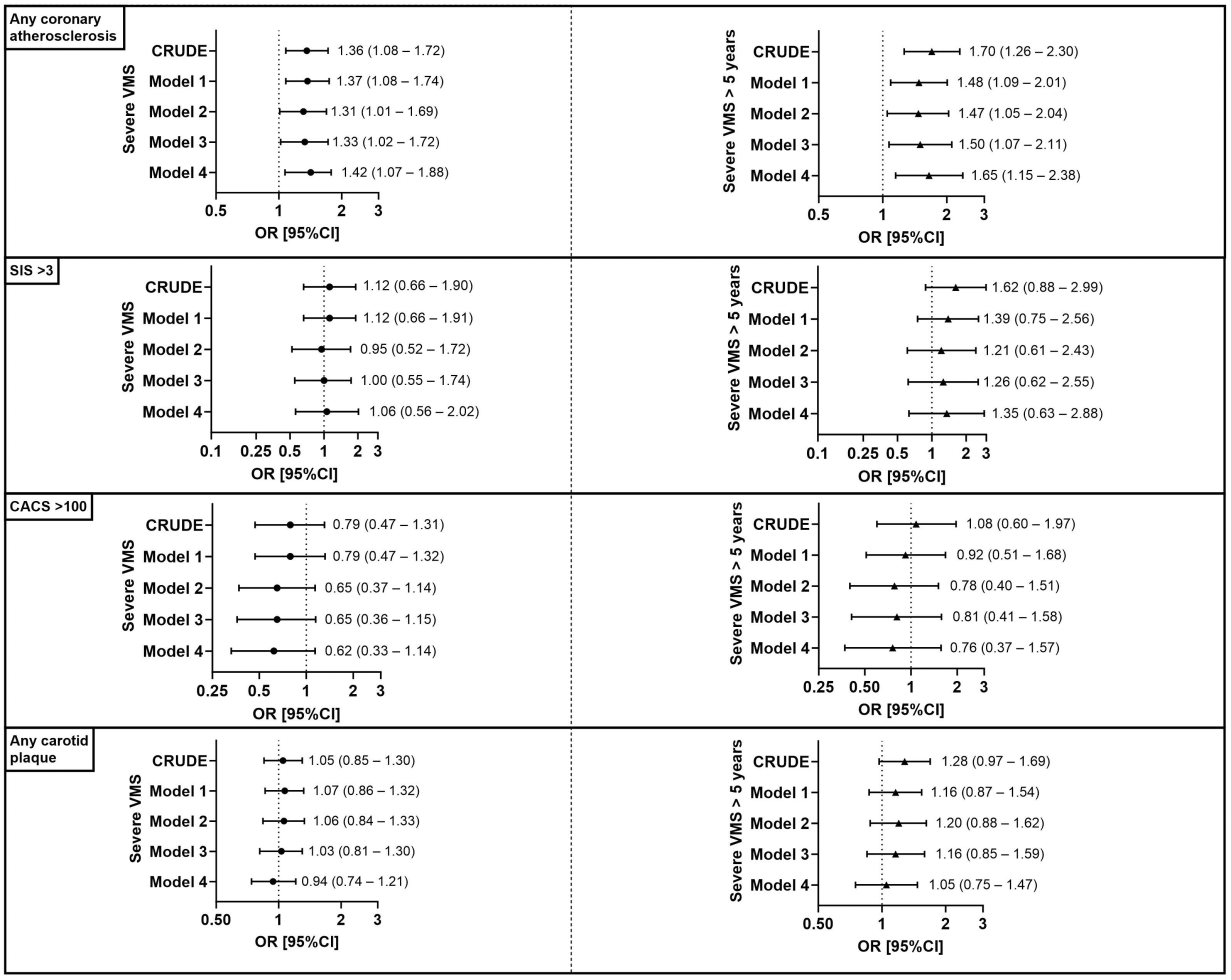
Associations between a history of severe vasomotor symptoms and the occurrence of subclinical ASCVD. A history of severe VMS (left panel) and severe VMS lasting >5 years (right panel) in relation to the occurrence of subclinical ASCVD, assessed in 4 different ways: any coronary atherosclerosis, SIS >3, CACS >100, and any carotid plaque. The logistic regression models underlying the results presented in the left panel include the exposure categories “ever mild/never VMS” (reference category), “ever moderate VMS,” and “ever severe VMS.” The results presented in the right panel derive from models including the exposure categories “ever mild/never VMS” (reference category), “ever moderate VMS lasting ≤5 years,” “ever moderate VMS lasting >5 years,” “ever severe VMS lasting ≤5 years,” and “ever severe VMS lasting >5 years.” Model 1 adjusts for age at time of study inclusion and site. Model 2 further adjusts for education, country of birth, menopausal status, systolic blood pressure, waist circumference, low‐density lipoprotein cholesterol, triglycerides, diabetes, hyperlipidemia, hypertension, smoking, and moderate to vigorous physical activity. Model 3 further adjusts for continuous stress, sleep quality, depression, and sleep apnea. Model 4 adjusts for the covariates included in model 3 and ever use of menopausal hormone therapy. ASCVD indicates atherosclerotic cardiovascular disease; CACS, coronary artery calcium score; OR, odds ratio; SCAPIS, Swedish Cardiopulmonary bioImage Study; SIS, segmental involvement score; and VMS, vasomotor symptoms.

For severe VMS that had lasted >5 years, the association with coronary atherosclerosis was more pronounced (crude OR, 1.70 [95% CI, 1.26–2.30]; Figure). This association was weakened but still statistically significant in models 1 through 3, whereas in model 4 it was again somewhat strengthened (Figure and Table S12). For severe VMS that had lasted ≤5 years, no significant association with coronary atherosclerosis was noted (Table S12). For moderate VMS, no effect of symptom duration was observed in relation to coronary atherosclerosis (Table S12).

For “ever severe VMS,” but not for “ever moderate VMS,” that started before the age at FMP, a significant association with coronary atherosclerosis was observed that persisted after multivariable adjustments, model 3 OR 1.66 (95% CI, 1.10–2.50) and model 4 OR 1.69 (95% CI, 1.11–2.58) (Table S13). Neither “ever severe VMS” nor “ever moderate VMS” starting at the age at FMP or later was significantly associated with coronary atherosclerosis (Table S13).

Analyses that used the “ever mild VMS group” as reference category, yielded similar associations with coronary atherosclerosis for both “ever moderate VMS” and “ever severe VMS” as the main analyses (Table S14). Results from analyses that included women with diagnosed ischemic heart disease were also not substantially different from these main results (Table S15).

Neither ever moderate nor ever severe VMS significantly associated with CACS >100, widespread coronary disease (SIS >3), or any carotid plaque (Figure and Table S10). Ever severe VMS were significantly associated with CACS >0 in the crude model but not after multivariable adjustments (Table S16).

## Discussion

In this population‐based study, we found that a self‐reported history of severe, but not moderate, VMS was significantly associated with coronary atherosclerosis detected with CCTA, independent of a wide range of lifestyle, socioeconomic, and clinical cardiovascular risk factors. To our knowledge this is the first time CCTA data have been used assessing the association between VMS and subclinical ASCVD.

In keeping with a previous study,^9^ women who ever experienced severe VMS generally had the longest duration of VMS. Interestingly, our results show that severe VMS lasting >5 years were significantly associated with coronary atherosclerosis, after multiple adjustments, whereas no corresponding association was observed for severe VMS of shorter durations. Moderate VMS, however, was not associated with coronary atherosclerosis regardless of such duration. To the best of our knowledge, no previous study of VMS in relation to subclinical atherosclerosis has addressed the importance of duration of VMS of different severity. However, the prospective American SWAN (Study of Women's Health Across the Nation) showed that the duration of frequent VMS (hot flashes or night sweats ≥6 days in a 2‐week period) was significantly linked to the risk of incident CVD; both analyses of continuous effect of the duration as well as effect when applying a cutoff corresponding to approximately 6 years of duration of frequent VMS were performed.^19^ The study did not address whether the duration of less frequent VMS was also linked to increased CVD risk. Our finding that pre‐FMP but not post‐FMP onset of severe VMS was significantly associated with coronary atherosclerosis is on par with findings from the SWAN study, which show an association between pre‐FMP onset of VMS and greater carotid intima media thickness after adjustments for demographic and cardiovascular risk factors.^18^ Our results are also in line with findings from the WISE (Women's Ischemia Syndrome Evaluation) showing associations between early‐onset VMS and reduced endothelial function and increased CVD mortality in a population of women referred for coronary angiography.^15^ However, results from a study that pooled data from 6 different prospective studies show that both premenopausal and postmenopausal onset of VMS are associated with incident CVD.^20^ It must be taken into account that the statistical precision of our results on post‐FMP onset of severe and moderate VMS, respectively, is low and no strong conclusions can therefore be drawn. The persisting association between severe VMS and coronary atherosclerosis after adjustment for conditions known to be prevalent in women with VMS, such as sleep disturbances and depression,^9^, ^11^ is on the one hand surprising as VMS could have a negative impact on lifestyle and sleep quality, which in turn could increase the risk of ASCVD. On the other hand, our results are consistent with several previous studies of VMS in relation to subclinical CVD and incident manifest CVD in which no substantial impact on the results was observed when adjusting for such covariates.^12^, ^13^, ^14^, ^19^, ^20^ Although it cannot be ruled out that severe VMS is merely a marker of an unfavorable cardiovascular risk profile, our findings of an independent relationship between severe VMS and coronary atherosclerosis could indicate an unknown underlying pathophysiological mechanism, either in the initiation or the progression of the atherosclerotic process in midlife women. Further research is required to elucidate mechanisms by which VMS may exert a deleterious effect on the coronary arteries, but we can speculate that women with long‐term severe VMS or whose severe VMS started early may have increased sensitivity to hormonal fluctuations, which in turn may contribute to atherosclerotic development via autonomic neurovascular dysregulation.^37^ Thus, these women may in general have an underlying adverse vascular reactivity manifested during the menopausal transition. Effects could perhaps be related to an overreactivity of the sympathetic nervous system, with inhibition of cardiac vagal control,^21^ or to changes in the hypothalamic–pituitary–adrenal axis associated with variable cortisol concentrations.^22^, ^23^ Furthermore, because the withdrawal of estrogen during menopausal transition rather than the absolute plasma level may be connected to an instability in the thermoregulatory center in the hypothalamus,^38^ it seems reasonable that increased central thermoregulatory sensitivity or hampered adaptation capacity may indirectly be related to a heightened central sympathetic tone, resulting in overactivity of the sympathetic nervous system.^38^, ^39^

The analyses of VMS in relation to the other subclinical ASCVD measurements—CACS >100, SIS >3, and any carotid plaque—showed no significant associations. To improve comparison to previous study results, we added analyses of VMS in relation to CACS >0. Still, we observed no significant associations after multiple adjustments (Table S12). These findings are in line with most previous studies on VMS in relation to CACS or carotid atherosclerosis^10^ with some exceptions.^12^, ^13^ CCTA is a diagnostic and noninvasive angiographic method able to detect coronary artery disease, compared with CAC that instead predicts risk for future manifest CVD events.^40^ Although CACS reflects the sum of coronary calcifications, CCTA can detect calcified as well as noncalcified plaques, degree of plaque stenosis, and extended plaque features such as plaque stability and anatomical distribution.^41^ Thus, VMS might be associated with early coronary atherosclerosis, before the development of calcified plaques, which might explain the observation of a significant and independent association with CCTA‐detected atherosclerosis that was not present with coronary calcification. Because SIS >3 was uncommon in the included age group, we might lack statistical power to detect significant differences and thus cannot rule out true associations. Although the atherogenesis appears to be similar for both coronary and carotid plaques,^42^ the lack of significant associations between severe VMS and any carotid plaque could relate to a different pathophysiology behind the development of coronary and carotid atherosclerosis.

For analyses of VMS in relation to ASCVD, it is not obvious which reference category should be used. Previous studies have mostly used women without VMS, whereas we also included women who reported current or previous mild VMS. We found that women who never experienced VMS had significantly more cardiovascular risk factors and coronary atherosclerosis than women with mild VMS, a finding consistent with the results from the WISE study, which observed an increased CVD mortality in this group compared with women with late‐onset VMS.^15^

Although the current study was not designed to study treatment effects, it is interesting to note that the association between >5 years of severe VMS and coronary atherosclerosis became stronger when adjusting for MHT, as this could possibly signal a mitigating effect. However, the corresponding influence on the OR point estimates for CACS >100 and carotid plaque was in the opposite direction. Further, this result must be interpreted with caution due to bias related to confounding by indication. Contraindications for prescribing MHT included a history of CVD, largely based on the results of the Women's Health Initiative,^43^ which means a selection of cardiovascular healthier women prescribed MHT. On the other hand, VMS severity could also influence whether the woman is treated with MHT or not, which, under the assumption that severe VMS affect the development of subclinical atherosclerosis, means a selection of less cardiovascular healthy women prescribed MHT.

### Study Limitations

There are several limitations to our study. First, the cross‐sectional observational study design limits any causal conclusions to be drawn because we do not know when VMS appeared in relation to the atherosclerosis development. There may also be residual confounding in our data; factors of possible relevance not accounted for include endogenous levels of estradiol and subtypes of MHT. Second, the results may not be generalizable to other geographical regions, races, ethnicities, and age groups because the definitions and prevalence of VMS differ.^6^, ^9^ Furthermore, generalization of the results should be made only to women with natural menopause. A limitation in this regard is that menopausal status was uncertain for women taking hormones. Third, the assessment of VMS may be subject to recall bias, given that up to 10 to 15 years could have passed since the study participants experienced their symptoms. In particular, a history of mild VMS may have been underreported. However, it is likely that such recall bias would be nondifferential because the women were not aware of their measures of subclinical coronary atherosclerosis or other cardiovascular risk assessments when they completed the questionnaire. In that case, this would have a weakening effect on our results. Finally, worth considering when interpreting the study results is that the assessment of VMS severity in this study was based on the woman's perceived level of discomfort, which means that caution should be used when comparing studies that objectively assess VMS.

### Clinical Perspectives and Need for Future Research

The findings of the current study, showing that women who suffer from severe VMS—especially if the symptoms are persistent and begin before the FMP—are more likely to have atherosclerosis in the coronary arteries than women who report no or only mild symptoms may contribute to strengthen the scientific evidence that these women should be extra carefully monitored for cardiovascular risk factors. However, further efforts are required to increase knowledge about potentially underlying pathophysiology. Increased knowledge is also needed about whether treatment with MHT can mitigate the potentially negative impact of VMS on cardiovascular risk. It seems important with multidisciplinary research approaches in this field.

## Conclusions

A history of severe VMS, with >5 years duration or beginning before the FMP, was significantly associated with subclinical CCTA‐detected atherosclerosis, independent of a wide range of socioeconomic, demographic, lifestyle‐related, and clinical cardiovascular risk factors. No corresponding associations were observed for SIS >3, CACS >100, or any carotid plaque. Further research is required to establish a possible causal relationship between severe VMS and coronary atherosclerosis. However, our findings support a more consistent implementation of preventive cardiovascular strategies across the health care system, targeting women with severe VMS, which may be a marker of poor or ongoing deterioration of cardiovascular health.

## Sources of Funding

The main funding body of the Swedish CardioPulmonary bioImage Study (SCAPIS) is the Swedish Heart‐Lung Foundation. The study is also funded by the Knut and Alice Wallenberg Foundation, the Swedish Research Council, Vinnova (Sweden's innovation agency), the University of Gothenburg, Sahlgrenska University Hospital, Karolinska Institutet, Stockholm County Council, Linköping University, Linköping University Hospital, Lund University, Skåne University Hospital, Umeå University, Umeå University Hospital, Uppsala University, and Uppsala University Hospital. The current study was supported by grants from the Swedish Heart‐Lung Foundation (grant number 20220190) to K.L. and grants from the County Council of Östergötland to A‐C.S.H. The funders had no role in the design and conduct of the study; collection, management, analysis, and interpretation of data; preparation, review, or approval of the article; and decision to submit the article for publication.

## Disclosures

None.

## Supporting information

Data
Tables S1–S16
Figure S1
References [44, 45, 46, 47]
