# Supplementary material for: Menopausal Vasomotor Symptoms and Subclinical Atherosclerotic Cardiovascular Disease: A Population‐Based Study
Source: J Am Heart Assoc. 2024 Aug 21;13(17):e033648. doi: 10.1161/JAHA.123.033648 (PMC11646512; doi:10.1161/JAHA.123.033648)
Supplement: Supplementary file 1 — Data Tables S1–S16 Figure S1 References [44, 45, 46, 47] [file JAH3-13-e033648-s001.pdf]

SUPPLEMENTAL MATERIAL

Data S1 Supplemental Methods.

Categorization of variables

- **Born outside Sweden**, yes/no, binary covariate
  Respondents were asked about whether Sweden was their country of birth.

- **University degree**, binary covariate
  The respondents were asked to indicate their highest level of education. Alternatives for highest completed level of education were: 1) elementary school or corresponding compulsory education, 2) different forms of high school education and 3) exam from university/college. A binary variable was created by merging alternatives 1 and 2 and letting this category form the reference.

- **Currently employed**, yes/no, binary covariate
  The respondents were asked about their current employment status. Whether the employment was full-time or part-time was not taken into account for the classification.

- **Financial strain**, yes/no, binary covariate
  Respondents were asked about difficulties managing regular expenses for food, rent, bills etc. the last 12 months.

- **Parental history of myocardial infarction and stroke**, yes/no, binary covariates
  Data on family history of myocardial infarction and stroke,

respectively, were obtained from the questionnaire as derived variables. A parental history of myocardial infarction was considered present if any parent had suffered a fatal or non-fatal myocardial infarction before 60 years of age. A parental history of stroke was considered present when any parent had suffered from stroke before 65 years of age.

- **Bad or very bad sleep quality**, binary covariate
  Respondents were asked how well they usually sleep.
  A 5-degree scale was used: 1. 'very bad', 2. 'bad', 3. 'normal', 4. 'good', 5. 'very good'. A binary variable was created by merging 'Very bad' and 'Bad' and letting the other categories in combination form the reference. The questions were adapted from the Basic Nordic Sleep Questionnaire[44].

- **Smoking status**, 3-level categorical covariate
  Responders were asked if they were current smokers, past smokers, or had never smoked.

- **High alcohol consumption**, binary covariate
  The questionnaire used the Alcohol Use Disorders Identification Test (AUDIT)[45] which consists of 10 separate questions about alcohol consumption. For each response, a certain number of points are assigned that add up to generate a total score. The total score categorizes each participant according to a 4-level scale: 1. 'low risk', 2. 'risky', 3. 'harmful' or 4. 'severe'. Here we collapsed the two highest levels ('harmful' and 'severe').

- **Continuous stress**, binary covariate
  The questionnaire used a 5-degree scale to identify experiences of stress: 1. ‘never experienced’, 2. ‘experienced some period of stress in life’, 3. ‘experienced some period of stress last 5 years’, 4. ‘experienced continuous stress last year’, 5. ‘experienced continuous stress last 5 years’). The definition of stress was feeling tense, irritable, anxious or having sleeping difficulties because of conditions at work or at home. Questions were adapted from previous studies investigating the association between stress and CVD[46]. The two highest levels (‘continuous stress last year’ and ‘continuous stress last 5 years’) were collapsed and contrasted against the other categories together.

- **Depression**, binary covariate
  The questionnaire used the short form of Composite International Diagnostic Interview of Diagnostic and Statistical Manual of Mental Disorders-IV to assess the presence of depressive symptoms the last 12 months, in concordance with recommendations from previous literature[47]. Depression was assessed by asking the respondent if she during the past twelve months had felt sad, blue, or depressed for two weeks or more in a row. If the answer was yes, seven following dichotomous questions were used to grade the depressive symptoms: lost interest in most things, feel tired or low on energy, gain or lose weight, trouble falling asleep, trouble concentrating,

think of death, feeling worthless. Present depression was defined as five or more positive responses.

- **Medical history**, yes/no, binary covariates
  Data on medical history were derived from four different sources: 1) self-reported in the core questionnaire, 2) International Classification of Diseases (ICD)-10, -9, and -8-codes from the National Patient Register (NPR), 3) Anatomical Therapeutic Chemical (ATC)-codes from the National Prescribed Drugs Register, and 4) biochemical assays related to SCAPIS data collection. The disease or group of diseases was defined as follows:

  - **Ischemic heart disease**
    Either of the following three criteria met: 1) Diagnosis of ischemic heart disease (angina pectoris excluded) in the NPR, either at an outpatient or inpatient clinic (ICD10 codes I21-I25; ICD9 and ICD8 codes 410-412 and 414), 2) Self-report in the core questionnaire of having undergone Coronary artery bypass graft (CABG) surgery or Percutaneous coronary intervention (PCI), 3) Observed coronary stent in the Coronary computed tomography angiography (CCTA) images.

  - **Hypertension**
    Either of the following two criteria met: 1) Self-reported hypertension in the core questionnaire, 2) Diagnosis of hypertension in the NPR, either at an

outpatient or inpatient clinic (ICD10 codes I10-I15; ICD9 codes 401-405; ICD8 codes 400-404) AND retrieved a medication for hypertension from a pharmacy (ATC codes C03 and C07-C09).

- **Hyperlipidemia**
  Either of the following two criteria met: 1) Self-reported hyperlipidemia in the core questionnaire, 2) Diagnosis of hyperlipidemia in the NPR, either at an outpatient or inpatient clinic (ICD10 code E78; ICD9 code 272; ICD8 codes 272 and 279) AND retrieved a medication for hyperlipidemia from a pharmacy (ATC code C10).

- **Ischemic stroke**
  Either of the following criteria met: 1) Self-reported ischemic stroke in the core questionnaire, 2) Diagnosis of ischemic stroke in the NPR, either at an outpatient or inpatient clinic (ICD10 codes I63-I65; ICD9 codes 433-434; ICD8 codes 423, 434 and 437).

- **Diabetes**
  Either of the following four criteria met: 1) Derived diabetes diagnosis from the core questionnaire (HbA1c >48 mmol/mol or fasting glucose >7,0 mmol/L), 2) Self-reported diabetes diagnosis in the core questionnaire or in medical interview at inclusion, 3) Retrieved medication for diabetes from a pharmacy (ATC code A10), 4) Diagnosis of

diabetes in the NPR, either at an outpatient or inpatient clinic (ICD10 codes E10-E15; ICD9 and ICD8 code 250).

- **Sleep apnea**
  Self-reported, via the core questionnaire, history of physician-diagnosed or surgically treated sleep apnea

- **Pre-eclampsia**
  Either of the following two criteria met: 1) Self-reported pre-eclampsia diagnosis in the core questionnaire, 2) Diagnosis of pre-eclampsia in the NPR, either at an outpatient or inpatient clinic (ICD10 codes O11 and O14-O15; ICD9 code 642; ICD8 codes 637 and 661).

- **Gestational hypertension**
  Diagnosis of gestational hypertension in the NPR, either at an outpatient or inpatient clinic (ICD10 code O13; ICD9 code 642).

- **Gestational diabetes**
  Self-reported history of gestational diabetes in the core questionnaire.

Table S1. General characteristics by menopausal status. The SWH population, SCAPIS.

Characteristics	SWH population n = 4465	Premenopausal n = 413 9.2%	Perimenopausal n = 366 8.2%	Postmenopausal n = 3686 82.6%
**Age**, mean (SD)	57.4 (4.4)	52.7 (2.4)	53.0 (2.1)	58.4 (4.1)
**Born outside Sweden**, n (%)	516 (11.7)	58 (14.1)	39 (10.8)	419 (11.5)
**University degree**, n (%)	2144 (48.7)	207 (61.0)	201 (55.8)	1736 (47.8)
**Currently employed**, n (%)	3749 (85.0)	377 (92.0)	337 (93.6)	3035 (83.3)
**Financial strain**, n (%)	169 (3.8)	18 (4.4)	17 (4.7)	134 (3.7)
**Smoking status**, n (%)				
Past	1678 (38.6)	111 (28.0)	106 (29.9)	1461 (40.7)
Current	472 (10.9)	26 (6.5)	35 (9.9)	411 (11.4)

**Pack years for cigarettes**, median (25th – 75th percentile)	11.3 (4.8 – 20.0)	9.0 (4.0 – 16.8)	10.8 (4.5 – 17.0)	11.3 (4.8 – 20.5)
**Medical history**, n (%)				
Ischemic heart disease	64 (1.4)	2 (0.5)	3 (0.8)	59 (1.6)
Ischemic stroke	89 (2.0)	11 (2.7)	7 (1.9)	71 (1.9)
Hypertension	804 (18.0)	45 (10.9)	48 (13.1)	711 (19.3)
Hyperlipidemia	299 (6.7)	8 (1.9)	8 (2.2)	283 (7.7)
Diabetes mellitus	167 (3.7)	15 (3.6)	10 (2.7)	142 (3.9)
Sleep apnea	98 (2.2)	4 (1.0)	3 (0.8)	91 (2.5)
**Parental history**, n (%)				
Myocardial infarction	327 (7.4)	26 (6.4)	26 (7.3)	275 (7.6)

Stroke	294 (6.7)	20 (4.9)	33 (9.2)	241 (6.6)
**Overweight (25-29.9 kg/m^2)**, n (%)	1611 (36.1)	150 (36.3)	131 (35.8)	1330 (36.1)
**Obese (≥30 kg/m^2)**, n (%)	867 (19.4)	82 (19.9)	74 (20.2)	711 (19.3)
**Waist circumference (cm)**, mean (SD)	89.0 (12.4)	87.4 (11.7)	89.2 (12.0)	89.1 (12.6)
**Blood pressure (mmHg)**, mean (SD)				
Systolic blood pressure	128 (18)	123 (18)	124 (17)	128 (18)
Systolic blood pressure >140, n (%)	1085 (24.3)	84 (20.3)	60 (16.4)	941 (25.5)
Diastolic blood pressure	81 (11)	79 (12)	80 (10)	80 (11)
Diastolic blood pressure >90, n (%)	895 (20.0)	83 (20.1)	67 (18.3)	745 (20.2)

**Cardiometabolic biomarkers**				
LDL cholesterol (mmol/L), mean (SD)	3.2 (0.9)	2.9 (0.8)	3.0 (0.8)	3.3 (0.9)
Triglycerides (mmol/L), mean (SD)	1.1 (0.6)	0.9 (0.5)	1.0 (0.5)	1.1 (0.6)
hsCRP (mg/L), median (25th – 75th percentile)	0.9 (0.6 – 2.0)	0.9 (0.6 – 1.8)	0.8 (0.6 – 1.8)	1.0 (0.6 – 2.1)
HbA1c (mmol/mol), mean (SD)	36.2 (5.4)	34.7 (5.2)	35.3 (4.3)	36.5 (5.5)
**Bad or very bad sleep quality**, n (%)	756 (17.4)	58 (14.3)	59 (16.7)	639 (17.8)
**Physical activity** (% of all wear time), mean (SD)				
Sedentary	51.6 (9.9)	52.1 (9.9)	53.1 (9.5)	51.4 (9.9)
Moderate/vigorous activity	6.6 (3.3)	6.8 (3.2)	6.5 (3.1)	6.6 (3.4)

**High alcohol consumption**, n (%)	418 (10.6)	40 (11.0)	32 (9.6)	346 (10.6)
**Continuous stress last 1-5 years**, n (%)	1118 (25.7)	99 (24.6)	106 (29.7)	913 (25.4)
**Depression**, n (%)	1025 (23.4)	103 (25.4)	101 (28.4)	821 (22.7)

Variable definitions are presented in Data S1.

SCAPIS, Swedish CArdioPulmonary bioImage Study; SWH, Survey of Women's Health; SBP, systolic blood pressure; DBP, diastolic blood pressure; LDL, low density lipoprotein; hsCRP, high sensitivity C-reactive protein; SD, standard deviation.

Table S2. Reproduction-related characteristics by menopausal status. The SWH population, SCAPIS

Characteristics	SWH population n = 4465	Premeno-pausal n = 413 9.2%	Perimeno-pausal n = 366 8.2%	Postmeno-pausal n = 3686 82.6%
**Age at menopause**, mean (SD)	-	-	-	49.1 (5.6)
**Time since menopause**, median (25th – 75th percentile)	-	-	-	9 (5 – 14)
**VMS**, n (%)*				
Ever severe	544 (13.4)	-	30 (8.2)	514 (13.9)
Ever moderate	682 (16.8)	-	58 (15.8)	624 (16.9)
Ever mild	1584 (39.1)	-	163 (44.5)	1421 (38.6)
Never	1045 (25.8)	-	103 (28.1)	942 (25.6)
Current	1502 (37.1)	-	202 (55.2)	1300 (35.3)
Previous	1119 (27.6)	-	30 (8.2)	1089 (29.5)
Age at start, mean (SD)	50.0 (6.4)	-	50.6 (6.1)	49.9 (6.4)
Duration† >5years	947 (23.4)	-	13 (3.6)	934 (25.3)
Duration† ≤5years	1660 (45.0)	-	218 (59.6)	1442 (39.1)

Duration†, years, median (25th – 75th percentile)	4 (2 – 8)	-	2 (1 – 3)	5 (3 – 8)
**Ever bothered by other menopausal symptoms**, n (%)*	1106 (27.3)	-	107 (30.6)	999 (28.7)
**Menopausal hormone therapy**				
Ever use, n (%)	623 (14.0)	-	28 (7.7)	595 (16.1)
Start age, mean (SD)	51.2 (4.9)	-	50.7 (2.9)	51.2 (5.0)
Duration‡, years, median (25th – 75th percentile)	4 (1 – 6)	-	1 (1 – 4)	4 (2 – 6)
**Ever usage of herbal medicine for VMS**, n (%)	346 (8.8)	-	30 (9.3)	316 (8.8)

**Medical obstetric history**, n (%)				
Preeclampsia	244 (5.5)	19 (4.6)	26 (7.1)	199 (5.4)
Gestational hypertension	91 (2.0)	11 (2.7)	14 (3.8)	66 (1.8)
Gestational diabetes	110 (2.5)	13 (3.2)	2 (0.6)	95 (2.6)

*Not considering premenopausal women.

†Considering reports of both current and previous VMS.

‡Considering both current and previous users.

SCAPIS, Swedish CArdioPulmonary bioImage Study; SWH, Survey of Women's Health; VMS, vasomotor symptoms; MHT, menopausal hormone therapy; SD, standard deviation.

Table S3. Prevalence of subclinical ASCVD detected by CT, CCTA or carotid ultrasound by menopausal status. The SWH population, SCAPIS

Measures	SWH population n = 4465	Premeno-pausal n = 413 9.2%	Perimeno-pausal n = 366 8.2%	Postmeno-pausal n = 3686 82.6%
**CACS** (AU), n (%)				
>0	1177 (27.1)	52 (13.0)	56 (15.7)	1069 (29.8)
>100	251 (5.8)	7 (1.8)	8 (2.2)	236 (6.6)
**CCTA,** n (%)				
Any coronary athero-sclerosis	1076 (26.1)	48 (12.6)	57 (16.9)	971 (28.6)
SIS >3	158 (3.8)	3 (0.8)	6 (1.8)	149 (4.4)
**Any carotid plaque,** n (%)	2027 (45.5)	126 (30.5)	134 (36.6)	1767 (48.1)

SCAPIS, Swedish CArdioPulmonary bioImage Study; SWH, Survey of Women's Health; ASCVD, atherosclerotic cardiovascular disease; AU, Agatston units; CT, computed tomography; CCTA, coronary computed tomography angiography, CACS, coronary artery calcium score; SIS, segment involvement score.

Table S4: General characteristics by ever VMS category, with separate reporting for the 'Never VMS' group. Selection from the SWH, SCAPIS

Characteristics		Ever VMS, n = 2265 75.6%		
	Never VMS n = 730 24.4%	Ever mild n = 1297 43.3%	Ever moderate n = 543 18.1%	Ever severe n = 425 14.2%
**Age**, mean (SD)	58.7 (4.3)‡	58.0 (4.1)	57.7 (4.3)	58.4 (4.1)
**Born outside Sweden**, n (%)	100 (13.9)*	130 (10.1)	53 (9.9)	74 (17.7)‡
**University degree**, n (%)	357 (49.7)*	655 (51.3)	285 (53.3)	176 (42.1)†
**Currently employed**, n (%)	569 (78.8)‡	1112 (86.8)	462 (86.4)	345 (82.3)*
**Financial strain**, n (%)	30 (4.2)*	32 (2.5)	18 (3.4)	28 (6.7)‡
**Smoking status**, n (%)				
Past	265 (37.3)	488 (38.6)	217 (40.9)	197 (48.0)‡
Current	81 (11.4)	128 (10.1)	68 (12.8)	55 (13.4)*
**Pack years for cigarettes**, median (25th – 75th percentile)	10.7 (4.8 – 20.0)	10.5 (4.3 – 19.5)	11.8 (5.0 – 21.0)	13.3 (5.5 – 21.0)*

**Medical history**, n (%)				
Ischemic heart disease	16 (2.2)	23 (1.8)	5 (0.9)	4 (0.9)
Ischemic stroke	24 (3.3)†	17 (1.3)	11 (2.0)	6 (1.4)
Hypertension	146 (20.0)*	207 (16.0)	93 (17.1)	80 (18.8)
Hyperlipidemia	69 (9.5)	97 (7.5)	31 (5.7)	24 (5.6)
Diabetes mellitus	39 (5.3)†	39 (3.0)	14 (2.6)	16 (3.8)
Sleep apnea	18 (2.5)	27 (2.1)	8 (1.5)	12 (2.8)
**Parental history**, n (%)				
Myocardial infarction	48 (6.7)	96 (7.5)	32 (6.0)	32 (7.7)
Stroke	42 (5.8)	84 (6.6)	30 (5.6)	40 (9.6)*
**Overweight (25-29.9 kg/m^2)**, n (%)	246 (33.7)	465 (35.9)	224 (41.3)*	181 (42.6)*
**Obese (≥30 kg/m^2)**, n (%),	172 (23.6)‡	207 (16.0)	83 (15.3)	79 (18.6)
**Waist circumference (cm)**, mean (SD)	90.7 (13.6)‡	88.1 (12.5)	88.8 (11.7)	89.6 (11.7)*
**Blood pressure (mmHg)**, mean (SD)				
Systolic blood pressure	129 (18)*	127 (18)	128 (18)	128 (19)
Systolic blood pressure >140, n (%)	195 (26.7)*	295 (22.7)	127 (23.4)	102 (24.0)

Diastolic blood pressure	81 (11)*	80 (11)	81 (10)	80 (11)
Diastolic blood pressure >90, n (%)	150 (20.6)	249 (19.2)	98 (18.0)	78 (18.4)
**Cardiometabolic biomarkers**				
LDL cholesterol (mmol/L), mean (SD)	3.2 (1.0)	3.3 (0.9)	3.3 (0.9)	3.3 (1.0)
Triglycerides (mmol/L), mean (SD)	1.1 (0.5)*	1.0 (0.5)	1.0 (0.5)	1.1 (0.7)†
hsCRP (mg/L)**,** median (25th – 75th percentile)	1.0 (0.6 – 2.4)‡	0.9 (0.6 – 1.9)	0.9 (0.6 – 1.9)	1.1 (0.6 – 2.2)†
HbA1c (mmol/mol), mean (SD)	37.0 (7.1)*	36.3 (5.1)	36.3 (3.9)	36.6 (5.7)
**Bad or very bad sleep quality**, n (%)	111 (15.6)	170 (13.4)	106 (20.1)‡	118 (28.9)‡
**Physical activity** (% of all wear time), mean (SD)				
Sedentary	51.5 (10.6)	51.7 (9.7)	51.4 (9.7)	50.0 (9.7)†
Moderate/vigorous activity	6.7 (3.5)	6.7 (3.3)	6.8 (3.3)	6.5 (3.6)
**High alcohol consumption**, n (%)	59 (9.4)	101 (8.7)	76 (15.4)‡	52 (12.3)*

**Continuous stress last 1-5 years**, n (%)	173 (24.4)	290 (22.9)	155 (29.2)‡	133 (32.6)‡
**Depression**, n (%)	146 (20.3)	259 (20.3)	146 (27.3)†	120 (29.2)‡

Premenopausal women not considered.

For all statistical comparisons, the ‘ever mild VMS group’ is used as the reference category.

*p<0.05

†p<0.01

‡p<0.001

SCAPIS, Swedish CArdioPulmonary bioImage Study; SWH, Survey of Women’s Health; VMS, vasomotor symptoms; SBP, systolic blood pressure; DBP, diastolic blood pressure; LDL, low density lipoprotein; hsCRP, high sensitivity C-reactive protein: SD, standard deviation.

Table S5. Reproduction-related characteristics by ever VMS category, with separate reporting for the 'Never VMS' group. Selection from the SWH, SCAPIS

Characteristics	Never VMS n = 730 24.4%	Ever VMS, n = 2265 75.6%		
		Ever mild n = 1297 43.3%	Ever moderate n = 543 18.1%	Ever severe n = 425 14.2%
**Age at menopause***, mean (SD)	51.9 (9.1)	51.7 (6.4)	51.9 (7.1)	52.4 (10.1)
**Time since menopaus***, mean (SD)	9 (5 – 13)	8 (5 – 11)	7 (4 – 11)	8 (5 – 12)
**VMS,** n (%)				
Current	-	642 (53.8)	296 (57.1)	235 (58.8)
Previous	-	551 (46.2)	222 (42.9)	165 (41.3)
Age at start of VMS, mean (SD)	-	50.5 (6.1)	49.9 (5.6)	49.4 (6.4)
Duration, years, median (25th – 75th percentile)	-	4 (2 – 6)	5 (3 – 8)	6 (3 – 10)
**Other menopausal symptoms**, n (%)	114 (15.6)	374 (28.8)	210 (38.9)	178 (41.9)
**Menopausal hormone therapy**				
Ever use, n (%)	55 (7.5)	167 (12.9)	172 (31.7)	214 (50.4)

Start, age, mean (SD)	51.9 (7.0)	51.5 (5.0)	51.4 (4.0)	50.9 (4.6)
Duration, years, median (25th – 75th percentile)	4 (1 – 7)	2 (1 – 7)	3 (1 – 5)	5 (2 – 7)
**Ever usage of of herbal medicine for VMS**, n (%)	13 (0.02)	97 (7.5)	85 (15.7)	85 (18.7)
**Medical obstetric history**, n (%)				
Preeclampsia	31 (4.2)	76 (5.9)	25 (4.6)	22 (5.2)
Gestational hypertension	13 (1.8)	30 (2.3)	11 (2.0)	4 (0.9)
Gestational diabetes	19 (2.6)	32 (2.5)	10 (1.9)	7 (1.7)

Premenopausal women not considered. For all statistical comparisons, the 'ever mild VMS group' is used as the reference category.

*Postmenopausal women considered only.

FMP, final menstrual period; SCAPIS, Swedish CArdioPulmonary bioImage Study; SWH, Survey of Women's Health; VMS, vasomotor symptoms; MHT, menopausal hormone therapy.

Table S6. Prevalence of subclinical ASCVD detected by CT, CCTA or carotid ultrasound by ever VMS category with separate reporting for the 'Never VMS' group. Selection from the SWH, SCAPIS

Measures	Never VMS<br>n = 730<br>24.4%	Ever VMS, n = 2265<br>75.6%		
		Ever mild<br>n = 1297<br>43.3%	Ever moderate<br>n = 543<br>18.1%	Ever severe<br>n = 425<br>14.2%
**CACS** (AU)**,** n (%)				
>0	223 (31.6)	355 (28.1)	137 (25.7)	143 (34.4)*
>100	67 (9.5)‡	68 (5.4)	25 (4.7)	24 (5.8)*
**CCTA,** n (%)				
Any coronary atherosclerosis	201 (30.8)*	315 (26.1)	130 (25.9)	135 (34.1)†
SIS>3	34 (5.2)	50 (4.1)	16 (3.2)	20 (5.1)
**Any carotid plaque,** n (%)	323 (44.4)	619 (47.8)	255 (47.2)	202 (47.6)

Premenopausal women not considered. For all statistical comparisons, the "ever mild" VMS group is used as the reference category.

*p<0.05

†p<0.01

‡p<0.001

SCAPIS, Swedish CArdioPulmonary bioImage Study; SWH, Survey of Women´s Health; VMS, vasomotor symptoms; ASCVD, atherosclerotic cardiovascular disease; AU, Agatston units; CT, computed tomography; CCTA, coronary computed tomography angiography; CACS, coronary artery calcium score; SIS, segment involvement score.

Table S7. Frequency of missing data for each variable by menopausal status. The SWH population, SCAPIS

Variable*, n (%)	SWH population n = 4465	Premenopausal n = 413 9.2%	Perimenopausal n = 366 8.2%	Postmenopausal n = 3686 82.6%
**Born outside Sweden**	52 (1.2)	3 (0.7)	6 (1.6)	43 (1.2)
**University degree**	65 (1.5)	7 (1.7)	6 (1.6)	52 (1.4)
**Currently employed**	52 (1.2)	3 (0.7)	6 (1.6)	43 (1.2)
**Financial strain**	55 (1.2)	5 (1.2)	6 (1.6)	44 (1.2)
**Smoking status**	121 (2.7)	16 (3.8)	11 (3.0)	94 (2.6)
**Pack years for cigarettes**	2315 (51.8)	276 (66.8)	225 (61.5)	1814 (49.2)
**Sleep apnea**	52 (1.2)	3 (0.7)	6 (1.6)	43 (1.2)
**Parental history**				
Myocardial infarction	63 (1.4)	6 (1.5)	8 (2.2)	49 (1.3)
Stroke	63 (1.4)	6 (1.5)	8 (2.2)	49 (1.3)
**BMI**	1 (0.0)	0 (0.0)	0 (0.0)	1 (0.0)
**Waist circumference**	2 (0.0)	0 (0.0)	0 (0.0)	2 (0.1)
**Blood pressure**	1 (0.0)	0 (0.0)	0 (0.0)	1 (0.0)

**Cardiometabolic biomarkers**				
LDL cholesterol	2 (0.0)	2 (0.5)	2 (0.5)	23 (0.6)
Triglycerides	5 (0.0)	1 (0.2)	1 (0.3)	3 (0.1)
hsCRP	5 (0.0)	1 (0.2)	1 (0.3)	3 (0.1)
HbA1c	13 (0.3)	2 (0.5)	1 (0.3)	10 (0.3)
**Sleep quality**	116 (2.6)	8 (1.9)	14 (3.8)	94 (2.6)
**Physical activity**	50 (1.1)	9 (2.2)	3 (0.8)	38 (1.0)
**Alcohol consumption**	511 (11.4)	50 (12.1)	34 (9.3)	427 (11.6)
**Continuous stress 1-5 last years**	117 (2.6)	10 (2.4)	9 (2.5)	98 (2.7)
**Depression**	82 (1.8)	8 (1.9)	10 (2.7)	64 (1.7)
**Postmenopausal status**	260 (7.1)	-	-	-
**Menopausal age**	-	-	-	261 (7.1)
**Age at start of VMS†**	189 (6.7)	-	19 (7.6)	170 (6.6)
**Duration of VMS†**	189 (7.2)	-	19 (7.6)	170 (6.6)
**Ever VMS**	197 (4.9)		12 (3.3)	185 (5.0)
**Current VMS**	386 (9.5)		31 (8.5)	355 (9.6)
**Ever bothered by other**	218 (5.4)	-	16 (4.4)	202 (5.5)

**menopausal symptoms**‡				
**Ever usage of herbal medicine for VMS**‡	104 (2.6)	-	4 (1.1)	100 (2.7)
**Gestational diabetes**	73 (1.6)	9 (2.2)	10 (2.7)	54 (1.5)
**CACS**	119 (2.6)	13 (3.1)	9 (2.5)	97 (2.6)
**Any coronary atherosclerosis**	349 (7.8)	33 (8.0)	28 (7.7)	289 (7.8)
**Any carotid plaque**	10 (0.2)	0 (0.0)	0 (0.0)	10 (0.3)

*There were no data missing for the variables age, ischemic heart disease, ischemic stroke, hypertension, hyperlipidemia, diabetes mellitus, VMS frequency last 2 weeks, MHT, preeclampsia, and gestational hypertension.

†Excluding premenopausal women and women who reported never having VMS.

‡Excluding premenopausal women. SCAPIS, Swedish CArdioPulmonary bioImage Study.

SWH, Survey of Women’s Health, LDL, low density lipoprotein; hsCRP, high sensitivity C-reactive protein, VMS, vasomotor symptoms; MHT, menopausal hormone therapy; ASCVD, atherosclerotic cardiovascular disease; CT, computed tomography; CCTA, coronary computed tomography angiography; CACS, coronary artery calcium score.

Table S8. General characteristics of the groups excluded from the SWH in the SCAPIS.

Characteristics	Total study population n=2995	Excluded group, total n = 2048	Excluded group[*] n=1470	Excluded group[†] n=578
**Age**, mean (SD)	57.7 (4.2)	56.4 (4.5)	55.3 (4.5)	57.4 (4.4)
**Born outside Sweden**, n (%)	357 (11.9)	286 (14.1)	159 (10.8)	127 (22.0)
**University degree**, n (%)	1473 (49.2)	953 (47.2)	671 (45.6)	282 (48.8)
**Currently employed,** n (%)	2488 (83.1)	1724 (84.9)	1260 (85.7)	463 (80.1)
**Financial strain,** n (%)	108 (3.6)	96 (4.7)	61 (4.1)	35 (6.1)
**Smoking status,** n (%)				
Past	1167 (39.0)	763 (38.2)	511 (34.8)	252 (43.6)
Current	332 (11.1)	219 (11.0)	140 (9.5)	79 (13.7)
**Medical history,** n (%)				
Ischemic heart disease	48 (1.6)	24 (1.2)	28 (1.9)	10 (1.7)
Ischemic stroke	58 (1.9)	35 (1.7)	31 (2.1)	4 (0.7)

Hypertension	526 (17.6)	394 (19.2)	296 (20.1)	130 (22.5)
**Overweight (25-29.9 kg/m^2)**, n (%)	1116 (37.3)	695 (34.0)	495 (33.7)	200 (34.6)
**Obese (≥30 kg/m^2)**, n (%)	541 (18.1)	447 (21.8)	326 (22.2)	121 (20.9)
**Waist circumference (cm)**, mean (SD)	89.1 (12.5)	88.9 (12.3)	88.8 (12.2)	89.4 (12.6)
**Blood pressure (mmHg)**, mean (SD)				
Systolic blood pressure	128 (18)	127 (18)	127 (18)	126 (18)
Diastolic blood pressure	80 (10)	80 (11)	81 (12)	79 (10)
**Cardiometabolic biomarkers**				
LDL cholesterol (mmol/L), mean (SD)	3.3 (0.9)	3.1 (0.9)	3.1 (0.9)	3.1 (0.9)
Triglycerides (mmol/L), mean (SD)	1.0 (0.6)	1.0 (0.6)	1.1 (0.7)	1.0 (0.5)
hsCRP (mg/L), median (25th – 75th percentile)	0.9 (0.4 – 1.9)	0.9 (0.6 – 2.0)	0.9 (0.4 – 1.7)	0.8 (0.4 – 2.0)
HbA1c (mmol/mol), mean (SD)	36.5 (5.6)	36.5 (5.6)	35.7 (4.9)	36.6 (5.3)

**Continuous stress last 1-5 years**, n (%)	751 (25.1)	512 (25.7)	336 (22.9)	145 (25.1)
**Depression**, n (%)	671 (22.4)	502 (25.0)	353 (24.0)	148 (25.6)

*Excluded due to loss of menstruation due to other causes than natural menopause, no data available on VMS, or premenopausal status. In this group, missing data is always <37, except for CRP where missing is n=881.

†Excluded due to missing data on menopausal status. In this group, missing data for the variables presented are always <20, except for CRP where missing values are 55.

SCAPIS, Swedish CArdioPulmonary bioImage Study; SWH, Survey of Women's Health; SBP, systolic blood pressure; DBP, diastolic blood pressure; LDL, low density lipoprotein; hsCRP, high sensitivity C-reactive protein; SD, standard deviation.

Table S9. Prevalence of subclinical ASCVD detected by CT, CCTA or carotid ultrasound by menopausal status. The groups excluded from the SWH in the SCAPIS

Measures	Total study population n=2995	Excluded group, total n=2048	Excluded group* n=1470	Excluded group† n=578
**CACS** (AU), n (%)				
>0	858 (28.6)	486 (24.6)	318 (21.6)	167 (28.9)
>100	184 (6.1)	109 (5.5)	67 (4.6)	42 (7.3)
**CCTA,** n (%)				
Any coronary atherosclerosis	781 (26.1)	451 (24.0)	295 (20.1)	156 (27.0)
SIS>3	120 (4.0)	70 (3.7)	39 (2.5)	31 (5.3)
**Any carotid plaque,** n (%)	1399 (46.7)	894 (43.6)	627 (42.6)	267 (46.1)

*Excluded due to loss of menstruation due to other causes than menopause, no data available on VMS, or premenopausal status. Number of missing values: CACS:44; CCTA:108; Any carotid plaque: 1

†Excluded due to missing data on menopausal status. Number of missing values: CACS:27; CCTA: 63; Any carotid plaque: 3

SCAPIS, Swedish CArdioPulmonary bioImage Study; SWH, Survey of Women's Health; ASCVD, atherosclerotic cardiovascular disease; AU, Agatston units; CT, computed tomography; CCTA, coronary computed tomography angiography, CACS, coronary artery calcium score; SIS, segment involvement score.

Table S10. Associations between severity of ever VMS and subclinical ASCVD. Selection from the SWH, SCAPIS

	OR (95% CI)		
	Ever mild/Never VMS *Reference category*	Ever moderate VMS	Ever severe VMS
**Any coronary atherosclerosis**	n = 1825 67.3%	n = 496 18.3%	n = 392 14.4%
CRUDE	-	0.94 (0.75 – 1.17)	1.36 (1.08 – 1.72)
Model 1	-	0.98 (0.78 – 1.24)	1.37 (1.08 – 1.74)
Model 2	-	1.01 (0.79 – 1.28)	1.31 (1.01 – 1.69)
Model 3	-	1.04 (0.81 – 1.32)	1.33 (1.02 – 1.72)
Model 4	-	1.07 (0.83 – 1.37)	1.42 (1.07 – 1.88)
**SIS >3**	n = 1825 67.3%	n = 496 18.3%	n = 392 14.4%
CRUDE	-	0.73 (0.41 – 1.28)	1.12 (0.66 – 1.90)
Model 1	-	0.77 (0.44 – 1.35)	1.12 (0.66 – 1.91)
Model 2	-	0.75 (0.41 – 1.34)	0.95 (0.52 – 1.72)
Model 3	-	0.70 (0.38 – 1.29)	1.00 (0.55 – 1.74)
Model 4	-	0.72 (0.39 – 1.35)	1.06 (0.56 – 2.02)
**CACS (AU) >100**	n = 1800 67.2%	n = 491 18.3%	n = 388 14.5%

CRUDE	-	0.79 (0.50 – 1.26)	0.79 (0.47 – 1.31)
Model 1	-	0.84 (0.53 – 1.33)	0.79 (0.47 – 1.32)
Model 2	-	0.79 (0.49 – 1.28)	0.65 (0.37 – 1.14)
Model 3	-	0.78 (0.48 – 1.27)	0.65 (0.36 – 1.15)
Model 4	-	0.76 (0.46 – 1.25)	0.62 (0.33 – 1.14)
**Any carotid plaque**	n = 1981<br>67.6%	n = 529<br>18.1%	n = 419<br>14.3%
CRUDE	-	1.03 (0.85 – 1.25)	1.05 (0.85 – 1.30)
Model 1	-	1.07 (0.88 – 1.30)	1.07 (0.86 – 1.32)
Model 2	-	1.03 (0.84 – 1.26)	1.06 (0.84 – 1.33)
Model 3	-	1.01 (0.82 – 1.24)	1.03 (0.81 – 1.30)
Model 4	-	0.97 (0.79 – 1.20)	0.94 (0.74 – 1.21)

Model 1: Adjustments for age at time of study inclusion + site; Model 2: Model 1 + highest degree of education, country of birth, systolic blood pressure, waist circumference, low density lipoprotein cholesterol, triglycerides, diabetes mellitus, hyperlipidemia, hypertension, smoking status, menopausal status, and moderate to vigorous physical activity; Model 3: Model 2 + continuous stress last 1-5 years, sleep quality, depression, and sleep apnea; Model 4: Model 3 + menopausal hormone therapy.

SCAPIS, Swedish CArdioPulmonary bioImage Study; SWH, Survey of Women's Health; VMS, vasomotor symptoms; ASCVD, atherosclerotic cardiovascular disease; AU, Agatston units; CCTA, coronary computed tomography angiography; CACS, coronary artery calcium score; SIS, segment involvement score; OR, odds ratio; CI, confidence interval.

Table S11. Association between severity of ever VMS and any coronary atherosclerosis after exclusion of late initiators of MHT (>6y after the age at final menstruation period). Selection from the SWH, SCAPIS

Models		OR (95 % CI)	
	Ever mild/Never VMS n = 1771 68.6%/ *Reference category*	Ever moderate VMS n = 454 17.6%	Ever severe VMS n = 357 13.8%
CRUDE	-	0.92 (0.72 – 1.16)	1.38 (1.08 – 1.76)
Model 1	-	0.98 (0.77 – 1.24)	1.38 (1.08 – 1.77)
Model 2	-	1.00 (0.78 – 1.29)	1.32 (1.02 – 1.72)
Model 3	-	1.02 (0.79 – 1.32)	1.35 (1.02 – 1.77)
Model 4	-	1.06 (0.82 – 1.37)	1.46 (1.09 – 1.95)

Model 1: Adjustments for age at time of study inclusion + site; Model 2: Model 1 + highest degree of education, country of birth, systolic blood pressure, waist circumference, low density lipoprotein cholesterol, triglycerides, diabetes mellitus, hyperlipidemia, hypertension, smoking status, menopausal status, and moderate to vigorous physical activity; Model 3: Model 2 + continuous stress last 1-5 years, sleep quality, depression, and sleep apnea; Model 4: Model 3 + menopausal hormone therapy.

SCAPIS, Swedish CArdioPulmonary bioImage Study; SWH, Survey of Women's Health; VMS, vasomotor symptoms; MHT, menopausal hormone therapy; OR, odds ratio; CI, confidence interval.

Table S12. Association between severity of ever VMS and any coronary atherosclerosis, considering VMS duration. Selection from the SWH, SCAPIS

Models	OR (95 % CI)				
	Ever mild/Never VMS n = 1825 67.3% *Reference category*	Ever moderate VMS ≤ 5 y n = 207 7.6%	Ever moderate VMS > 5 y n = 289 10.7%	Ever severe VMS ≤ 5y n =192 7.1%	Ever severe VMS > 5y n = 200 7.4%
CRUDE	-	0.87 (0.65 – 1.16)	1.03 (0.75 – 1.43)	1.06 (0.76 – 1.48)	1.70 (1.26 – 2.30)
Model 1	-	1.07 (0.79 – 1.43)	0.89 (0.64 – 1.23)	1.25 (0.89 – 1.76)	1.48 (1.09 – 2.01)
Model 2	-	1.12 (0.82 – 1.53)	0.88 (0.62 – 1.24)	1.14 (0.80 – 1.64)	1.47 (1.05 – 2.04)
Model 3	-	1.16 (0.85 – 1.59)	0.89 (0.63 – 1.27)	1.14 (0.78 – 1.66)	1.50 (1.07 – 2.11)
Model 4	-	1.18 (0.87 – 1.62)	0.94 (0.66 – 1.35)	1.19 (0.82 – 1.75)	1.65 (1.15 – 2.38)

Model 1: Adjustments for age at time of study inclusion + site; Model 2: Model 1 + highest degree of education, country of birth, systolic blood pressure, waist circumference, low density lipoprotein cholesterol, triglycerides, diabetes mellitus,

hyperlipidemia, hypertension, smoking status, menopausal status, and moderate to vigorous physical activity; Model 3: Model 2 + continuous stress last 1-5 years, sleep quality, depression, and sleep apnea; Model 4: Model 3 + menopausal hormone therapy.

SCAPIS, Swedish CArdioPulmonary bioImage Study; SWH, Survey of Women's Health; VMS, vasomotor symptoms; OR, odds ratio; CI, confidence interval.

Table S13. Association between severity of ever VMS and any coronary atherosclerosis, taking into account age at onset of VMS in relation to age at final menstrual period. Selection from the SWH, SCAPIS

Model	OR (95 % CI)				
	Ever mild/Never VMS n = 1660 *Reference category*	Ever moderate VMS with pre-FMP onset n = 184	Ever moderate VMS with post-FMP onset n = 254	Ever severe VMS with pre-FMP onset n =155	Ever severe VMS with post-FMP onset n = 206
CRUDE	-	0.97 (0.69 – 1.39)	0.93 (0.68 – 1.27)	1.53 (1.05 – 2.33)	1.34 (0.98 – 1.84)
Model 1	-	1.03 (0.72 – 1.49)	0.98 (0.71 – 1.35)	1.60 (1.09 – 2.35)	1.36 (0.93 – 1.88)
Model 2	-	1.06 (0.73 – 1.55)	0.98 (0.71 – 1.37)	1.58 (1.06 – 2.37)	1.29 (0.95 – 1.81)
Model 3	-	1.12 (0.76 – 1.63)	0.98 (0.70 – 1.37)	1.66 (1.10 – 2.50)	1.30 (0.92 – 1.85)
Model 4	-	1.13 (0.77 – 1.66)	0.99 (0.71 – 1.31)	1.69 (1.11 – 2.58)	1.33 (0.93 – 1.90)

Pre-FMP onset was defined as VMS onset before the age at FMP. Post-FMP onset was defined as VMS onset at FMP or older.

Model 1: Adjustments for age at time of study inclusion + site; Model 2: Model 1 + highest degree of education, country of birth, systolic blood pressure, waist circumference, low density lipoprotein cholesterol, triglycerides, diabetes mellitus, hyperlipidemia, hypertension, smoking status, menopausal status, and moderate to vigorous physical activity; Model 3: Model 2 + continuous stress last 1-5 years,

sleep quality, depression, and sleep apnea; Model 4: Model 3 + menopausal hormone therapy.

SCAPIS, Swedish CArdioPulmonary bioImage Study; SWH, Survey of Women's Health; VMS, vasomotor symptoms; OR, odds ratio, CI, confidence interval.

Table S14. Association between ever VMS and any coronary atherosclerosis using the 'Ever mild VMS group,' as reference. Selection from the SWH, SCAPIS

Model	OR (95 % CI)			
	Ever mild VMS n = 1186 43.7% *Reference category*	Never VMS n = 639 23.6%	Ever moderate VMS n = 496 18.3%	Ever severe VMS n = 392 14.4%
CRUDE	-	1.26 (1.02 – 1.56)	1.02 (0.80 – 1.29)	1.48 (1.16 – 1.90)
Model 1	-	1.17 (0.94 – 1.45)	1.04 (0.81 – 1.33)	1.45 (1.13 – 1.87)
Model 2	-	1.13 (0.90 – 1.43)	1.05 (0.81 – 1.36)	1.37 (1.05 – 1.79)
Model 3	-	1.11 (0.87 – 1.40)	1.07 (0.83 – 1.39)	1.38 (1.04 – 1.81)
Model 4	-	1.10 (0.87 – 1.39)	1.11 (0.85 – 1.44)	1.46 (1.09 – 1.96)

Model 1: Adjustments for age at time of study inclusion + site; Model 2: Model 1 + highest degree of education, country of birth, systolic blood pressure, waist circumference, low density lipoprotein cholesterol, triglycerides, diabetes mellitus, hyperlipidemia, hypertension, smoking status, menopausal status, and moderate to vigorous physical activity; Model 3: Model 2 + continuous stress last 1-5 years, sleep quality, depression, and sleep apnea; Model 4: Model 3 + menopausal hormone therapy.

SCAPIS, Swedish CArdioPulmonary bioImage Study; SWH, Survey of Women's Health; VMS, vasomotor symptoms; OR, odds ratio, CI, confidence interval.

Table S15. Association between severity of ever VMS and any coronary atherosclerosis after inclusion of women with previously diagnosed ischemic heart disease. Selection from the SWH, SCAPIS

Models		OR (95 % CI)	
	Ever mild/Never VMS<br>n = 1858<br>*Reference category*	Ever moderate VMS<br>n = 501	Ever severe VMS<br>n = 396
CRUDE	-	0.91 (0.73 – 1.14)	1.35 (1.07 – 1.70)
Model 1	-	0.96 (0.76 – 1.21)	1.35 (1.07 – 1.71)
Model 2	-	0.99 (0.78 – 1.25)	1.30 (1.01 – 1.68)
Model 3	-	1.01 (0.79 – 1.29)	1.31 (1.01 – 1.70)
Model 4	-	1.04 (0.82 – 1.34)	1.40 (1.06 – 1.86)

Model 1: Adjustments for age at time of study inclusion + site; Model 2: Model 1 + highest degree of education, country of birth, systolic blood pressure, waist circumference, low density lipoprotein cholesterol, triglycerides, diabetes mellitus, hyperlipidemia, hypertension, smoking status, menopausal status, and moderate to vigorous physical activity; Model 3: Model 2 + continuous stress last 1-5 years, sleep quality, depression, and sleep apnea; Model 4: Model 3 + menopausal hormone therapy.

SCAPIS, Swedish CArdioPulmonary bioImage Study; Survey of Women's Health; VMS, vasomotor symptoms; MHT, menopausal hormone therapy; OR, odds ratio; CI, confidence interval.

Table S16. Association between severity of ever VMS and CACS (AU) >0. Selection from the SWH, SCAPIS

Models	OR (95 % CI)		
	Ever mild/Never VMS<br>n = 1800<br>67.2%<br>*Reference category*	Ever moderate VMS<br>n = 491<br>18.3%	Ever severe VMS<br>n = 388<br>14.5%
CRUDE	-	0.88 (0.70 – 1.10)	1.34 (1.06 – 1.69)
Model 1	-	0.91 (0.73 – 1.15)	1.35 (1.06 – 1.71)
Model 2	-	0.93 (0.72 – 1.18)	1.25 (0.97 – 1.62)
Model 3	-	0.94 (0.74 – 1.21)	1.24 (0.95 – 1.61)
Model 4	-	0.98 (0.76 – 1.26)	1.33 (1.00 – 1.77)

Model 1: Adjustments for age at time of study inclusion + site; Model 2: Model 1 + highest degree of education, country of birth, systolic blood pressure, waist circumference, low density lipoprotein cholesterol, triglycerides, diabetes mellitus, hyperlipidemia, hypertension, smoking status, menopausal status, and moderate to vigorous physical activity; Model 3: Model 2 + continuous stress last 1-5 years, sleep quality, depression, and sleep apnea; Model 4: Model 3 + menopausal hormone therapy.

SCAPIS, Swedish CArdioPulmonary bioImage Study; SWH, Survey of Women's Health; VMS, vasomotor symptoms; AU, Agaston units; CACS, coronary artery calcium score; OR, odds ratio; CI, confidence interval.

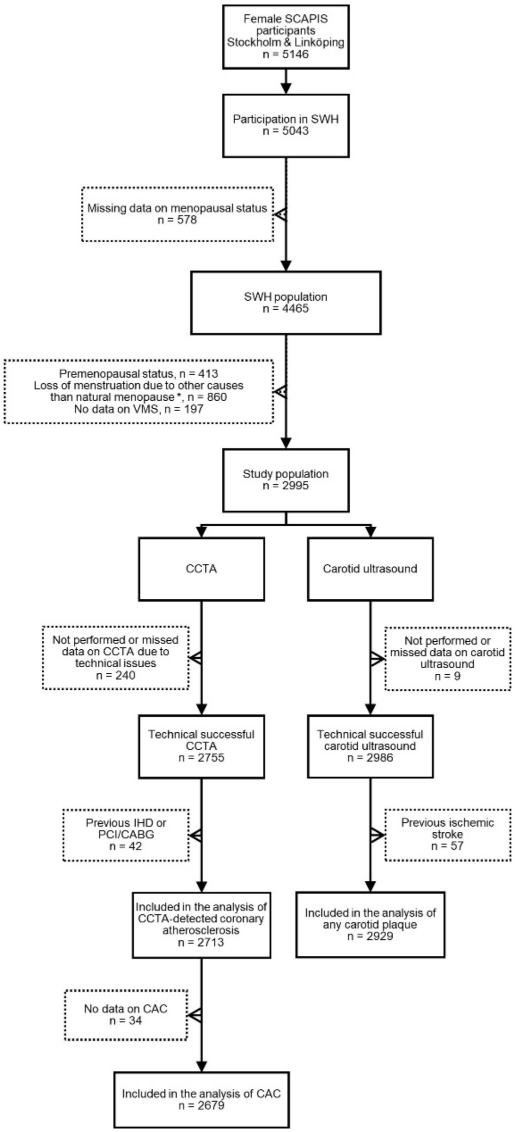

Figure S1. Selection of the study population.

*Hormonal treatment, surgical menopause, excessive exercising, dietary restrictions, pregnancy or breastfeeding.

ASCVD, atherosclerotic cardiovascular disease; CABG, Coronary Artery Bypass Graft CAC, Coronary Artery Calcium; CCTA, Coronary Computed Tomography Angiography; IHD, Ischemic Heart Disease; PCI, Percutaneous Coronary

Intervention; SCAPIS, Swedish CArdioPulmonary bioImage Study; SWH, Survey of Women's Health; VMS, vasomotor symptoms.
